# Characterization of *Aspergillus* species on Brazil nut from the Brazilian Amazonian region and development of a PCR assay for identification at the genus level

**DOI:** 10.1186/1471-2180-14-138

**Published:** 2014-05-30

**Authors:** Glaucia EO Midorikawa, Maria de Lourdes M de Sousa, Otniel Freitas Silva, Jurema do Socorro A Dias, Luis IB Kanzaki, Rogerio E Hanada, Renata MLC Mesquita, Rivadalve C Gonçalves, Virginia S Alvares, Daniela MC Bittencourt, Robert NG Miller

**Affiliations:** 1 Departamento de Biologia Celular, Instituto de Ciências Biológicas, Universidade de Brasília, Campus Universitário Darcy Ribeiro, CEP 70.910-900 Brasília D.F., Brazil; 2 EMBRAPA Centro Nacional de Pesquisa de Tecnologia Agroindustrial de Alimentos, Avenida das Americas, 29501, Guaratiba, CEP 23.020-470 Rio de Janeiro, RJ, Brazil; 3 EMBRAPA Amapa, Rodovia Juscelino Kubitschek, Km 5, No. 2600, CEP 68.903-419 Caixa Postal 10, Macapa, AP, Brazil; 4 Instituto Nacional de Pesquisas da Amazônia, Avenida André Araújo, 2936, CEP 69.067-375, Caixa Postal 2223, Manaus, AM, Brazil; 5 EMBRAPA Acre, BR 364, Km 14, Zona Rural, CEP 69.908.970, Caixa Postal 321, Rio Branco, AC, Brazil; 6 EMBRAPA Amazônia Ocidental, Rodovia AM-010, Km 29, CEP 69.011-970, Caixa Postal 319, Manaus, AM, Brazil

**Keywords:** *Aspergillus* section *Flavi*, Mycotoxin, Mitochondrial small subunit ribosomal RNA gene, PCR-RFLP

## Abstract

**Background:**

Brazil nut is a protein-rich extractivist tree crop in the Amazon region. Fungal contamination of shells and kernel material frequently includes the presence of aflatoxigenic *Aspergillus* species from the section *Flavi*. Aflatoxins are polyketide secondary metabolites, which are hepatotoxic carcinogens in mammals. The objectives of this study were to identify *Aspergillus* species occurring on Brazil nut grown in different states in the Brazilian Amazon region and develop a specific PCR method for collective identification of member species of the genus *Aspergillus.*

**Results:**

Polyphasic identification of 137 *Aspergillus* strains isolated from Brazil nut shell material from cooperatives across the Brazilian Amazon states of Acre, Amapá and Amazonas revealed five species, with *Aspergillus* section *Flavi* species *A. nomius* and *A. flavus* the most abundant. PCR primers ASP_GEN_MTSSU_F1 and ASP_GEN_MTSSU_R1 were designed for the genus *Aspergillus*, targeting a portion of the mitochondrial small subunit ribosomal RNA gene. Primer specificity was validated through both electronic PCR against target gene sequences at Genbank and in PCR reactions against DNA from *Aspergillus* species and other fungal genera common on Brazil nut. Collective differentiation of the observed section *Flavi* species *A. flavus*, *A. nomius* and *A. tamarii* from other *Aspergillus* species was possible on the basis of RFLP polymorphism.

**Conclusions:**

Given the abundance of *Aspergillus* section *Flavi* species *A. nomius* and *A. flavus* observed on Brazil nut, and associated risk of mycotoxin accumulation, simple identification methods for such mycotoxigenic species are of importance for Hazard Analysis Critical Control Point system implementation. The assay for the genus *Aspergillus* represents progress towards specific PCR identification and detection of mycotoxigenic species.

## Background

Aflatoxins (AF) are polyketide family secondary metabolites produced by several members of the fungal genus *Aspergillus,* section *Flavi*. Considered amongst the most dangerous natural hepatotoxic carcinogens in mammals [1], consumption of foodstuffs contaminated with these extrolites can be a cause of mortality and reduced productivity in higher vertebrates. Within this family, AFB1, B2, G1 and G2 cause most concern, given their abundance and toxicity [2]. The mycotoxin cyclopiazonic acid (CPA) [3] can also be produced by aspergilli. This toxic indole tatramic acid is associated with damage to liver, heart and kidneys [4].

The taxonomy of the genus *Aspergillus* is complex, with overlapping morphological characteristics and biochemical properties between species, as well as intraspecific polymorphism [5,6]. *Aspergillus* section *Flavi* comprises over 20 member species, based on polyphasic approaches for species delimitation that consider morphological, molecular and extrolite data [7-10]. A number of species within the section are aflatoxigenic, including the widely distributed species *A. flavus*, *A. parasiticus* and *A. nomius*, together with *A. arachidicola*, *A. bombycis*, *A. minisclerotigenes*, *A. parvisclerotigenus*, *A. pseudocaelatus, A. pseudonomius* and *A. pseudotamarii,* ([7] and references therein), *A. novoparasiticus*[8], *A. mottae, A. sergii* and *A. transmontanensis*[9].

Brazil nut (*Bertholletia excelsa* Humb. & Bompl.) is a protein-rich oily nut, which, as an extractivist tree crop, provides employment to communities in the Amazon region. Currently, Bolivia and Brazil are the world’s largest producers, with annual production in excess of 40 thousand tons [11]. Aflatoxin contamination negatively affects exports, with maximum tolerable limits imposed by the European Commission of 8.0 μg/kg and 5.0 μg/kg for AFB1, for unshelled and shelled nuts, respectively, and 15.0 μg/kg and 10.0 μg/kg for total aflatoxins (AFB1, AFB2, AFG1 and AFG2). *A. flavus* and *A. nomius* are common aflatoxin producers on Brazil nut [12,13], with less frequent isolation of aflatoxigenic species *A. arachidicola*, *A. bombycis*, *A. parasiticus* and *A. pseudotamarii*[12,14,15]. Non aflatoxigenic species include *Flavi* section members *A. caelatus* and *A. tamarii*, as well as aspergilli which are not classified in the section, such as *A. versicolor* and *A. sydowii*[12].

Given that morphological characters can be insufficient for distinguishing certain species belonging to section *Flavi*, numerous molecular-based approaches have been developed. These have included analysis of rDNA ITS and aflR-aflJ intergenic spacers for differentiation of *A. flavus* and *A. parasiticus*[16,17], as well as AFLP and SNP analysis for differentiation of *A. flavus/A. oryzae*, *A. parasiticus/A. sojae*, *A. tamarii* and *A. nomius*[18,19]. Sequence-based approaches include analysis of rDNA ITS and 28S rRNA variable regions [20,21], together with calmodulin and β-tubulin gene regions [7,22,23]. Variability in the latter two genes can be appropriate for resolving closely related *Aspergillus* species [24]. Molecular identification of nine species of section *Flavi* was recently described, based upon amplification of aflT and aflR genes and rDNA ITS regions, genomic DNA *SmaI*-derived RFLPs, and RAPD fingerprinting [25]. Specific detection of section *Flavi* species in contaminated material has been described using both PCR e.g. [26] and loop-mediated isothermal amplification [27].

Hazard Analysis Critical Control Point (HACCP) methods are employed to reduce the risk of contamination of foods with microbial pathogens, toxins or allergens [28]. When setting up HACCP concepts, species identification is necessary for determining critical control points (CCPs) in the field, storage or transport. In this context, the objectives of this study were to identify *Aspergillus* species occurring on Brazil nut from different states in the Brazilian Amazon region on the basis of morphological, molecular and extrolite data, followed by the development of a PCR method for collective identification of member species of the genus *Aspergillus*.

## Results

### Identification and abundance of *Aspergillus* species

Polyphasic identification of all 137 *Aspergillus* strains isolated from Brazil nut shell material collected from cooperatives across the Brazilian Amazon region (states of Acre, Amapá and Amazonas) revealed the presence of five species, with three belonging to *Aspergillus* section *Flavi*. Blastn-derived analyses against *Aspergillus* species sequences deposited in Genbank for ex-type strains revealed similarities of between 99 and 100% for rDNA ITS, β-tubulin and calmodulin sequences.

Qualitative analysis of mycotoxigenic potential in representative strains of the aflatoxigenic species isolated from different regions revealed, for *A. flavus*, AFB1, AFB2 and CPA production in 11 evaluated strains, and AFB1 and CPA production for a further five strains. From a total of seven examined strains of *A. nomius*, five produced AFB1, AFB2, AFG1 and AFG2, one produced B1 and G1, and one produced B1, G1 and G2. CPA was not detected in *A. nomius*.

When considering totals for each species from all growing areas analysed, aflatoxigenic species *A. nomius* and *A. flavus* were the most abundant, representing 43.1 and 42.3% of all isolated aspergilli, respectively (Table 1). The non aflatoxigenic species *A. tamarii* was observed at a lower overall frequency (13.13%). *Aspergillus* species which do not belong to section *Flavi* were also isolated, with one isolate of *A. fumigatus* from Amapá and one isolate of *A. niger* from Amazonas. When comparing *A. nomius* and *A. flavus*, although similar numbers of strains were identified in total, numbers varied considerably across regions, with *A. nomius* more frequent in samples from Amapá, Coari (Amazonas), Itacoatiara (Amazonas) and Manicoré (Amazonas), and *A. flavus* more common in contaminated material from Acre and Humaitá (Amazonas).

**Table 1:** **Frequency of *Aspergillus *** **species from Brazil nut material across the Brazilian Amazon region**

**State**	**Number of strains isolated from Brazil nut material**				
	***A. nomius***	***A. flavus***	***A. fumigatus***	***A. tamarii***	***A. niger***
Acre	1 (5.3)*	18 (94.7)	0	0	0
Amapá	20 (95.2)	0	1 (4.8)	0	0
Amazonas					
Coari	5 (83.3)	0	0	1 (16.7)	0
Humaitá	7 (14.3)	40 (81.6)	0	1 (2.05)	1 (2.05)
Itacoatiara	19 (90.5)	0	0	2 (9.5)	0
Manicoré	7 (33.33)	0	0	14 (66.66)	0
**Total**	59 (43.1)	58 (42.3)	1 (0.73)	18 (13.13)	1 (0.73)

*Values in parentheses indicate percentages for each species for each geographical region.

#### MtDNA primer development for genus

**Figure 1:**
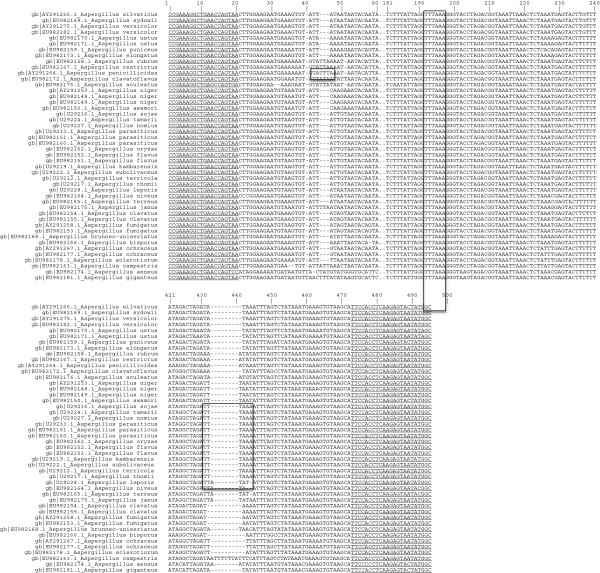
**Nucleotide sequence alignment of a portion of the mtDNA SSU rRNA among *Aspergillus *species.** Sequences available in Genbank were downloaded from NCBI. Underlined sequences indicate annealing positions for the specific primers ASP_GEN_MTSSU_F1 and ASP_GEN_MTSSU_R1. Boxed sequences indicate *Dra*I restriction sites.

Following sequence alignment of a portion of the mtDNA SSU rRNA gene from Genbank-derived sequences for all available *Aspergillus* species, specific primers ASP_GEN_MTSSU_F1 (5′-GCCATATTACTCTTGAGGTGGAA-3′) and ASP_GEN_MTSSU_R1 (5′-CCGAAAGGCTGAACCAGTAA-3′) were designed for amplification of a 480 bp PCR product specific for the genus (Figure 1). *In silico* analysis of the specificity of the primer pair was based upon electronic PCR against mtDNA SSU rDNA gene sequences available at Genbank for the genus *Aspergillus* and fungi from additional genera previously reported on Brazil nut [29]. Positive nucleotide BLAST search results with 0% mismatch were observed against target mtDNA SSU rRNA from all available *Aspergillus* species, as well as teleomorphs from the genera *Chaetosartorya*, *Emericella*, *Eurotium* and *Petromyces*. For all additional fungal genera documented on this host, primer annealing sites were absent in sequences. Wider testing across all deposited fungal mitochondrial DNA sequences in Genbank revealed primer target sequences in *Mycena* sp., *Monascus purpureus* and *Leiothecium ellipsoideum*, although expected amplicon sizes were at least 41 bp shorter than that expected for the genus *Aspergillus*.

**Figure 2:**
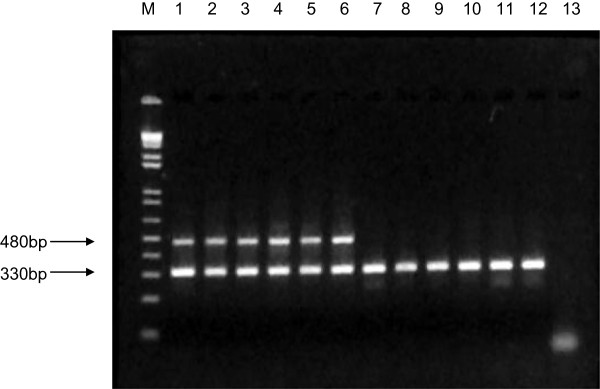
**PCR amplification of a specific mtDNA SSU rRNA amplicon for species members of the genus *Aspergillus *using primers ASP_GEN_MTSSU_F1 and ASP_GEN_MTSSU_R1, together with co-amplification of an internal amplification control.** M: 1 Kb plus DNA ladder; 1–2: *Aspergillus flavus*; 3: *Aspergillus nomius*; 4: *Aspergillus tamarii*; 5: *Aspergillus fumigatus*; 6: *Aspergillus niger*; 7–8: *Fusarium solani* f. sp. *glycines*; 9: *Fusarium solani*; 10: *Penicillium citrinum*; 11: *Trichoderma harzianum*; 12: *Cladosporium cladosporioides*; 13: negative control.

When validating specificity of the primer pair against fungal DNA, a PCR product of the expected size was amplified only from members of the genus *Aspergillus*, with no amplification observed for other fungal genera associated with *B. excelsa* (Figure 2). An IAC was included for co-amplification in each sample to prevent false negative results which could potentially be caused by PCR inhibitors [30]. An IAC concentration of 10 pg was identified as optimum for simultaneous amplification of the 480 bp specific *Aspergillus* amplicon and the 330 bp IAC with primers ASP_GEN_MTSSU_F1, ASP_GEN_MTSSU_R1 and M13 reverse. Validation of the specific primers for detection of *Aspergillus* DNA directly from naturally contaminated samples showed that amplification of the genus-specific PCR product was possible from a minimum of 10 ng of total DNA extracted from Brazil nut material.

### RFLP analysis

Restriction maps for the specific mtDNA SSU rRNA amplicon for the genus were compared across the *Aspergillus* species isolated from Brazil nut. Minor nucleotide sequence differences were detected, with the restriction endonuclease *Dra*I appropriate for differentiating the isolated *Aspergillus* section *Flavi* members from other species in the genus also encountered on Brazil nut. According to the restriction maps for the five isolated *Aspergillus* species in this study, two conserved restriction sites are present for this enzyme in the target amplicon region for the isolated *Aspergillus* section *Flavi* members *A. flavus*, *A. nomius* and *A. tamarii*, which should result in PCR product cleavage into fragments of 30, 170 and 237 bp. Predicted restriction digest patterns were compared in mtDNA SSU rRNA sequences available in Genbank for section *Flavi* species *A. parasiticus*, *A. oryzae* and *A. sojae*, together with the *A. flavus* synonyms *A. kambarensis, A. subolivaceus and A. thomii*[7]), and for the *A. tamarii* synonym *A. terricola*[7]). These sequences showed the same two conserved *Dra*I restriction sites, in contrast to distinct RFLP profiles observed in sequences for *Aspergillus* species not belonging to section *Flavi* (Additional file 1), as well as in the *Aspergillus* teleomorphs and non-target genera *Mycena*, *Monascus* and *Leiothecium*.

**Figure 3:**
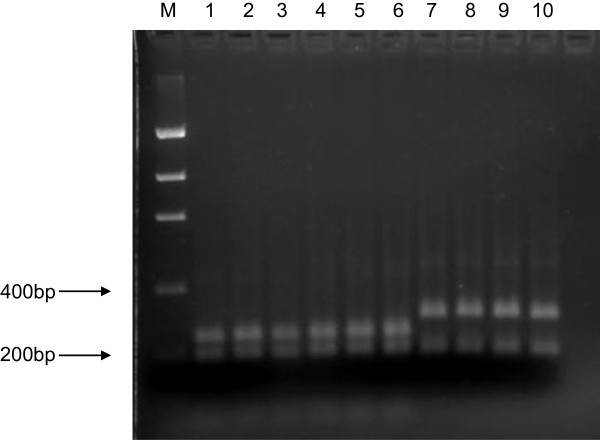
***Dra*I restriction digest profiles of the specific mtDNA SSU rRNA amplicon for differentiation of *Aspergillus *section *Flavi *species members from other aspergilli.** M: Low DNA Mass Ladder; 1–3: *Aspergillus flavus*; 4–5: *Aspergillus nomius*; 6: *Aspergillus tamarii*; 7–8: *Aspergillus fumigatus*; 9–10: *Aspergillus niger*.

In order to validate the restriction mapping data, PCR RFLP analysis was conducted on PCR-amplified specific mtDNA SSU rRNA amplicons across the different *Aspergillus* species isolated. PCR-RFLPs with *Dra*I confirmed differentiation of these three section *Flavi* members from the other *Aspergillus* species, with digest patterns in agreement with *in silico* data (Figure 3).

## Discussion

Morphology-based methods for identification of species of the genus *Aspergillus* can be unreliable as a result of both intraspecific similarities and differences [16]. In this present study, identification of *Aspergillus* species on Brazil nut from different states in the Brazilian Amazon region was conducted according to Samson and Varga [6] and Baquião et al. [14], through morphological and molecular characterization, together with extrolite profile (aflatoxins and CPA). As observed in previous studies for section *Flavi*[24,31], species identifications based upon analyses of rDNA ITS, β-tubulin and calmodulin gene sequence identities against sequences for ex-type strains available through the NCBI nucleotide nr database provided results in agreement with morphology-based identification and extrolite production.

The frequency we observed of aflatoxigenic *Aspergillus* section *Flavi* species from Brazil nut shell material confirmed recent reports that *A. nomius* and *A. flavus* are abundant species on Brazil nut across production areas in the Brazilian Amazonian region [14,32]. In our study, these two species represented over 85% of all *Aspergillus* species isolated. Qualitative analysis of mycotoxin production in strains of the mycotoxigenic species representative of the different states of origin supported the identifications, with *A. flavus* strains producing AFB and CPA, and *A. nomius* producing AFB and AFG, without CPA production. The extrolite profiles are in agreement with expected chemical characterization data for these member species in the section [16,33].

Given the documented widespread occurrence of both *A. flavus* and *A. nomius* on Brazil nut, together with the known capacity to produce mycotoxins AFB and CPA, and AFB and AFG, respectively, the presence of these species on husk materials represents a threat to safe production of Brazil nut. As the section *Flavi* species *A. tamarii* and *A. fumigatus* are also documented producers of CPA [34,35], the occurrence of these species on Brazil nut highlights the need for regulations which also consider this mycotoxin.

PCR-based molecular diagnosis of microorganisms offers specificity and sensitivity appropriate for early detection, appropriate for both HACCP purposes [36] and implementation of countermeasures for control of microbial contamination. As Brazil nut is an extractivist crop, with aflatoxigenic species occurring throughout the production chain [32,37], safe production is dependent upon identification of CCPs and subsequent implementation of detection methods at these points. The mitochondrial genome is an attractive molecule for application in fungal taxonomy and systematics, with a rapid rate of evolution and limited genetic recombination [38,39]. For *Aspergillus*, both specific and intraspecific level comparisons have been described [40,41]. Considering the high copy number per cell, mitochondrial DNA (mtDNA) is also easily amplifiable by PCR and appropriate for characterization through RFLP analysis. In the current study, analysis of the mtDNA SSU rRNA gene region enabled the design of a genus-specific primer pair for amplification of a 480 bp PCR product in *Aspergillus*. Specific amplification was possible using DNA extracted from pure cultures, as well as from naturally contaminated Brazil nut samples. Together with the developed IAC, this PCR-based method has potential for inclusion in the setup of HACCP concepts. Many attempts with genetic markers for differentiation of section members at the interspecific level have not provided sufficient resolution for detection of small differences across the fungal genomes. In the case of the closely related species *A. flavus* and *A. oryzae*, minor differences across the genome can only be revealed by detecting differences across numerous loci, such as digestion of total DNA with restriction endonucleases [42] or aflatoxin biosynthetic pathway gene interspecific polymorphism [43]. Similarly, the closely related species *A. parasiticus* and *A. sojae* can only be distinguished using genetic markers such as RAPD [44]. Our approach based upon the use of genus specific primers for mtDNA SSU rDNA followed by RFLPs appeared to resolve phylogenetically distant species, with the three section *Flavi* member species encountered in this study all displaying a single RFLP profile. *In silico* analysis of restriction sites in the target mtDNA SSU rDNA sequence for all *Aspergillus* species available in Genbank supported the observed polymorphisms delimiting in a group specific manner, separating section *Flavi* species from other species not classified in the section. Further investigation of this polymorphism is warranted across all member species of the section.

## Conclusions

In conclusion, five species of *Aspergillus* were identified on Brazil nut material from cooperatives across states in the Brazilian Amazon region, with the aflatoxigenic *Aspergillus* section *Flavi* species *A. nomius* and *A. flavus* the most abundant. A specific PCR-based method for identification at the genus level was developed*,* which also enabled collective differentiation of the observed section *Flavi* species *A. flavus*, *A. nomius* and *A. tamarii* from other *Aspergillus* species, on the basis of RFLP polymorphism. Given the widespread distribution of *Aspergillus* section *Flavi* species and associated risk of food contamination due to mycotoxin accumulation, simple molecular methods to aid identification of mycotoxigenic species are of importance in identification of CCPs at the point of production and storage, from which appropriate management practices can be developed.

## Methods

### Fungal isolation

Strains belonging to the genus *Aspergillus* were isolated from 3 L samples of Brazil nut collected from cooperatives in growing areas in eastern and western regions of the Brazilian Amazon (Amapá, Amazonas and Acre states). A total of three localities were sampled per state. Isolation into pure culture from shell tissues was performed according to Freire et al. [45]. Single spore cultures were used throughout the study, with all strains preserved both in 20% glycerol at – 80°C and on silica gel at 4°C. Strains were identified to species level based on macroscopic colony morphology and conidial morphology, extrolite production, and sequence data identities for rDNA ITS, β-tubulin and calmodulin gene regions, as described previously [7,32,46]. A representative isolate for each haplotype of each identified *Aspergillus* species was preserved as a single spore culture and deposited in the reference mycological culture collection at the Department of Phytopathology, University of Brasilia.

### Determination of aflatoxins and cyclopiazonic acid

Analysis of mycotoxigenic potential of a number of *Aspergillus* section *Flavi* strains representative of each state was conducted under permissive conditions according to Schmidt-Heydt et al. [47], following growth at 25°C for 7 days on YES medium (20 g/L yeast extract, 150 g/L sucrose, 0,5 g/L MgSO_4_ 5H_2_O, 0.1 g de ZnSO_4_, 0.05 g CuSO4,15 g/L agar), with water activity adjusted to 0.99, using a glycerol/water mixture of 108 mL glycerol per litre. Aflatoxin and cyclopiazonic acid standards were acquired from Sigma-Aldrich (Saint Louis, MO, USA), with liquid chromatography grade solvents from Merck (Darmstadt, Germany). For each fungal colony, mycotoxins from the entire content for each colonized plate were extracted under shaking conditions in 10 mL methanol at room temperature for 60 min. Following simple filtration using Whatman No. 1 filter papers, 500 μL of type 1 purified H_2_O was added to 500 μL of supernatant and filtered through a 0.22 μm teflon membrane. A total of 10 μL of filtrate were diluted with 990 μL of acetonitrile:water (20:80, v/v). The filtrate (10 μL) was then subjected to UPLC/MS/MS analysis. Calibration curves were prepared for each mycotoxin standard using six concentrations: AFB1 0.25, 0.5, 1.0, 5.0, 7.5 and 10.0 ng/mL; AFB2 0.06, 0.125, 0.25, 1.25, 1.875, 2.50; AFG1 0.25, 0.50, 1.0, 5.0, 7.6, 10.0 ng/mL; AFG2 0.06, 0.125, 0.25, 1.25, 1.875, 2.50; ACP 5, 10, 20, 100, 150, 200 ng/mL). The R2 varied between 0.94 and 0.994, depending on the toxin. The quantification limits were 0.1 ng/mL for AFB1, 0.04 for AFB2, 0.10 for AFG1, 0.02 for AFG2 and 0.2 for CPA. Analyses were performed on an ACQUITY UPLC™ separation system coupled with a Quattro Premier™ XE tandem quadrupole mass spectrometer (Waters, Manchester, UK). The software MassLynx version 4.1 with application manager software QuanLynx (Waters) was employed for instrument control and data analysis. Chromatographic separation of toxins was conducted using an ACQUITY UPLC BEH C18 (1.7 μm, 2.1 × 100 mm; Waters). Elution was performed using the gradient: mobile phase A (H_2_O + 0.2% formic acid) and mobile phase B (acetonitrile + 0.2% formic acid): 0–1 min (10% B); 10 min (50% B); 10.5 min (85% B); 11 min (10% B); and 12 min (10% B). Flow rate was set at 0.4 mL/min, with a column temperature of 40ºC and total run time of 12 min. A full loop injection mode was employed, with an injection volume of 10 μL. The mass spectrometer was operated in mode with electronspray-ionization (ESI) source. Operating conditions were optimized as follows: capillary voltage, 3.5 kV (positive mode); ion source temperature, 120°C; desolvation temperature, 450°C; cone gas flow, 50 L/h; desolvation gas flow, 700 L/h (nitrogen gas in both cases); and collision gas flow, 0.15 mL/min (argon gas).

### Total DNA extraction

Cultures for each strain were grown on Czapek Yeast Autolysate agar (CYA) [46] for seven days at 25°C. Mycelial discs were subcultured into 150 mL of CYA liquid media and incubated for a further three days at 25°C, with agitation at 120 rev min^−1^. Mycelia were harvested by washing under sterile distilled water, vacuum filtration and freeze drying. Genomic DNA was extracted from 50 mg samples of macerated mycelia, as well as from naturally contaminated Brazil nut material, according to Raeder and Broda [48]. DNA was electrophoresed in 1% agarose gels at 5 V cm^−1^ in the presence of ethidium bromide (1 μg mL^−1^), with Low DNA Mass ladder® (Invitrogen) employed for quantification under UV at 254 nm.

### Molecular-based identification

For all the isolates characterized in this study, a fragment of each of the rDNA ITS1–5.8S–ITS2 region, the β-tubulin and calmodulin genes were amplified using the universal primers ITS5/ITS4 [49], T1/T22 [23], and cmd5/cmd6 [50], respectively. Each PCR reaction contained 10 ng of template DNA, 0.4 μM of each primer, 200 μM dNTPs, 1.5 mM MgCl_2_, 1.0 U Taq DNA polymerase and 1× IB Taq polymerase buffer (Phoneutria, Belo Horizonte, MG, Brazil). Temperature cycling was conducted with the following program: denaturation at 95°C for 4 min, 30 cycles of denaturation at 94°C for 1 min, primer annealing at 50°C for 1 min, and extension at 72°C for 1 min, plus a final elongation period at 72°C for 5 min. PCR products were purified using ExoSAP-IT® (USB, Cleveland, Ohio, USA) and forward and reverse- sequenced using the Big Dye® Terminator v3.1 Cycle Sequencing kit (Applied Biosystems, Foster City, CA, USA). Products were run on an ABI 3700 DNA sequencer (Applied Biosystems, Foster City, CA, USA). Sequences were quality-edited and mounted into contigs using the program Sequencher, version 4.8 (Gene codes Corporation, Ann Arbor, MI USA). Strains were identified on the basis of sequence similarity using the program BLASTn [51], against both the NCBI nucleotide nr database and a local database of sequences for *Aspergillus* ex-type strains (Additional file 2).

Nucleotide sequences for unique haplotypes of each species were deposited in the NCBI database. Ribosomal DNA ITS1–5.8S–ITS2 sequences were deposited in Genbank with the accession numbers KJ634089, KJ634090, KJ634091, KJ634092 and KJ634093, β-tubulin gene sequences with accession numbers KJ634094, KJ634095, KJ634096 and KJ634097, and calmodulin gene sequences with accession numbers KJ634098 and KJ634099.

### mtDNA SSU rDNA characterization and primer design for the Genus

Based upon sequence alignment using ClustalW [52] of representative mtDNA SSU rDNA sequences for *Aspergillus* species available at Genbank® (http://www.ncbi.nlm.nih.gov/) (Additional file 3), specific primers for the genus ASP_GEN_MTSSU_F1 and ASP_GEN_MTSSU_R1 were designed using the software Primer3 [53]. In order to test primer specificity *in silico*, electronic PCR was conducted using the program primersearch, available through The European Molecular Biology Open Software Suite (EMBOSS). Based upon BLAST searches, the specific primers were tested against both the NCBI nucleotide database and a local database of mtDNA SSU rDNA gene sequences for fungi documented on Brazil nut [29,45], comprising members of the genera *Aspergillus*, *Acremonium, Chaetomium, Cladosporium, Colletotrichum, Exophiala, Fusarium, Graphium, Hypocrea, Paecilomyces, Penicillium, Phialophora, Phoma, Rhizopus* and *Trichoderma* (Additional file 3).

Specificity of the primer pair was validated in PCR reactions against DNA from *Aspergillus* species and other fungal genera common on Brazil nut [29], namely *A. flavus, A. nomius, A. tamarii, A. fumigatus, A. niger, Fusarium solani, Penicillium citrinum, Trichoderma harzianum,* and *Cladosporium cladosporioides*. PCR reactions were conducted using 15 ng of template fungal DNA together with 0.20 μM of each primer, 0,2 μg/μL of bovine serum albumin (BSA), 1.0U Taq DNA polymerase (Phoneutria, Belo Horizonte, MG, Brazil) and 1× IB Taq polymerase buffer (Phoneutria, Belo Horizonte, MG, Brazil). Validation was also performed on total DNA samples extracted from naturally contaminated Brazil nut samples, with a detection limit assessed on diluted DNA. PCR thermocycling, product purification, sequencing and editing were as described earlier, with an annealing temperature of 60°C. All experiments were conducted in duplicate, with a positive internal amplification control (IAC) present in each sample and a separate negative control lacking template DNA included with PCR amplifications. The specific PCR product amplified with primers ASP_GEN_MTSSU_F1 and ASP_GEN_MTSSU_R1 was firstly digested using the restriction enzyme SnaBI (New England BioLabs, Ipswich, MA, USA), and a 154 bp fragment containing the annealing site for primer ASP_GEN_MTSSU_F_1_ then cloned into the vector pGEMTeasy (Promega, Madison, WI, USA) according to standard protocols. Following cloning, a recombinant strain was stored as a glycerine culture at −80°C. Plasmid DNA was isolated and 10 pg used as an IAC template in all PCR reactions, together with the pGEM®-T Easy-targeting reverse primer M13, annealing to position 176 on the pGEM®-T Easy plasmid vector DNA sequence.

### mtDNA SSU rDNA PCR RFLP analysis

The potential of the mtDNA SSU rRNA gene amplicon for inter-specific differentiation was investigated based upon polymorphism in restriction sites. The target mtDNA sequence was analysed in each of the *Aspergillus* species available at Genbank, which included six *Aspergillus* species with complete mitochondrial genome sequences [54]*.* Restriction maps were generated for each species using the program Sequence Manipulation Suíte (http://www.bioinformatics.org/sms2/rest_map.html). Following identification of suitable restriction sites for differentiation, RFLP digestion of the specific mtDNA amplicons was then tested across the section *Flavi* species and additional *Aspergillus* species isolated from Brazil nut. Each digest reaction volume of 30 μL contained 1 mg of PCR product, 1 × restriction enzyme buffer React 1 (Invitrogen, Carlsbad, CA, USA), and 1 U of the selected restriction enzyme *Dra*I (Invitrogen, Carlsbad, CA, USA). Following a two hour incubation period at 37°C, digest fragments were electrophoresed in 1% agarose gels at 5 V cm^−1^ in the presence of ethidium bromide (1 μg mL^−1^), and visualized under UV at 254 nm. The marker Low DNA Mass ladder® (Invitrogen, Carlsbad, CA, USA) was included on gels for digest fragment size estimation.

## Competing interests

The authors declare that they have no competing interests.

## Authors’ contributions

GEOM participated in DNA extraction, polyphasic identification, sequencing and analysis, primer development and validation and RFLP analysis. MLMS participated in mycotoxin determination. OFS participated in mycotoxin determination. JSAD participated in collection of contaminated Brazil nut and fungal isolation. LIBK participated in collection of contaminated Brazil nut and fungal isolation. REH participated in collection of contaminated Brazil nut and fungal isolation. RMLCM participated in collection of contaminated Brazil nut, fungal isolation and molecular-based identification. RCG participated in collection of contaminated Brazil nut and fungal isolation. VSA conceived the study, participated in collection of contaminated Brazil nut and fungal isolation. DMCB conceived the study, participated in collection of contaminated Brazil nut, fungal isolation and molecular-based identification. RNGM conceived the study, participated in DNA extraction, polyphasic identification, sequencing and analysis, primer development and validation, RFLP analysis and drafted the manuscript. All authors have contributed to, read and approved the final manuscript.

## Supplementary Material

Additional file 1 **MtDNA SSU rRNA gene *Dra*** **I restriction mapping data for *Aspergillus *** **species.** Click here for file

Additional file 2 **Ribosomal DNA ITS, beta-tubulin and calmodulin gene sequences deposited at Genbank for *Aspergillus *** **ex-type strains.** Click here for file

Additional file 3 MtDNA SSU rRNA gene sequences deposited at Genbank for fungi documented on Brazil nut. Click here for file
