# Supplementary material for: Characterization of Aspergillus species on Brazil nut from the Brazilian Amazonian region and development of a PCR assay for identification at the genus level
Source: BMC Microbiol. 2014 May 30;14:138. doi: 10.1186/1471-2180-14-138 (PMC4051963; doi:10.1186/1471-2180-14-138)
Supplement: Additional file 1 — MtDNA SSU rRNA gene Dra I restriction mapping data for Aspergillus species. [file 1471-2180-14-138-S1.docx]

| **Species/NCBI ID** | **mtDNA SSU rRNA amplicon size (bp)** | ***Dra*I restriction site positions** | **Fragment sizes (bp)** |
| --- | --- | --- | --- |
| Aspergillus flavus gb\|EU982152.1 | 437 | 170, 407 | 30, 170, 237 |
| Aspergillus flavus gb\|EU982152.1 | 437 | 170, 407 | 30, 170, 237 |
| Aspergillus nomius gb\|U29227.1 | 437 | 170, 407 | 30, 170, 237 |
| Aspergillus parasiticus gb\|EU982160.1 | 437 | 170, 407 | 30, 170, 237 |
| Aspergillus parasiticus gb\|EU982161.1 | 437 | 170, 407 | 30, 170, 237 |
| Aspergillus parasiticus gb\|U29233.1 | 437 | 170, 407 | 30, 170, 237 |
| Aspergillus oryzae gb\|EU982162.1 | 437 | 170, 407 | 30, 170, 237 |
| Aspergillus sojae gb\|U29216.1 | 437 | 170, 407 | 30, 170, 237 |
| Aspergillus tamarii gb\|U29224.1 | 437 | 170, 407 | 30, 170, 237 |
| Aspergillus kambarensis gb\|U29219.1 | 437 | 170, 407 | 30, 170, 237 |
| Aspergillus subolivaceus gb\|U29222.1 | 437 | 170, 407 | 30, 170, 237 |
| Aspergillus thomii gb\|U29217.1 | 437 | 170, 407 | 30, 170, 237 |
| Aspergillus terricola gb\|U29212.1 | 437 | 170, 407 | 30, 170, 237 |
|  |  |  |  |
| Aspergillus leporis gb\|U29228.1 | 437 | 170 | 170, 267 |
| Aspergillus aculeatus gb\|EU982176.1 | 437 | 170 | 170, 267 |
| Aspergillus aeneus gb\|EU982174.1 | 440 | 172 | 172, 268 |
| Aspergillus awamori gb\|EU982150.1 | 437 | 170 | 170, 267 |
| Aspergillus bisporus gb\|EU982166.1 | 433 | 169 | 169, 264 |
| Aspergillus brunneo-uniseriatus gb\|EU982168.1 | 439 | 171 | 171, 268 |
| Aspergillus campestris gb\|EU982163.1 | 446 | 171 | 171, 275 |
| Aspergillus clavatoflavus gb\|EU982172.1 | 439 | 173 | 173, 266 |
| Aspergillus clavatus gb\|EU982154.1 | 435 | 170 | 170, 265 |
| Aspergillus clavatus gb\|EU982155.1 | 435 | 170 | 170, 265 |
| Aspergillus elongatus gb\|EU982173.1 | 436 | 170 | 170, 266 |
| Aspergillus fumigatus gb\|AY291258.1 | 435 | 267 | 168, 267 |
| Aspergillus fumigatus gb\|EU982153.1 | 435 | 170 | 170, 265 |
| Aspergillus giganteus gb\|EU982181.1 | 440 | 174 | 174, 266 |
| Aspergillus janus gb\|EU982175.1 | 441 | 173 | 173, 268 |
| Aspergillus niger gb\|AY291253.1 | 436 | 269 | 167, 269 |
| Aspergillus niger gb\|EU982148.1 | 437 | 170 | 170, 267 |
| Aspergillus niger gb\|EU982149.1 | 437 | 170 | 170, 267 |
| Aspergillus niveus gb\|EU982164.1 | 436 | 170 | 170, 266 |
| Aspergillus ochraceus gb\|AY291267.1 | 435 | 267 | 168, 267 |
| Aspergillus ochraceus gb\|EU982177.1 | 435 | 170 | 170, 265 |
| Aspergillus penicillioides gb\|AY291264.1 | 439 | 269, 415 | 24, 146, 269 |
| Aspergillus puniceus gb\|EU982159.1 | 436 | 172 | 172, 264 |
| Aspergillus restrictus gb\|EU982167.1 | 436 | 172 | 172, 264 |
| Aspergillus rubrum gb\|EU982158.1 | 439 | 172 | 172, 267 |
| Aspergillus sclerotiorum gb\|EU982178.1 | 435 | 170 | 170, 265 |
| Aspergillus silvaticus gb\|AY291266.1 | 437 | 269 | 168, 269 |
| Aspergillus sydowii gb\|EU982169.1 | 437 | 170 | 170, 267 |
| Aspergillus terreus gb\|EU982165.1 | 437 | 170 | 170, 267 |
| Aspergillus ustus gb\|EU982170.1 | 436 | 170 | 170, 266 |
| Aspergillus ustus gb\|EU982171.1 | 436 | 170 | 170, 266 |
| Aspergillus versicolor gb\|AY291275.1 | 437 | 269 | 168, 269 |
| Aspergillus versicolor gb\|EU982182.1 | 437 | 170 | 170, 267 |
| Eurotium herbariorum gb\|AY291259.1 | 439 | 269 | 170, 269 |
| Eurotium rubrum gb\|AF346424.1 | 438 | 171 | 171, 267 |
