# Supplementary material for: Characterization of Aspergillus species on Brazil nut from the Brazilian Amazonian region and development of a PCR assay for identification at the genus level
Source: BMC Microbiol. 2014 May 30;14:138. doi: 10.1186/1471-2180-14-138 (PMC4051963; doi:10.1186/1471-2180-14-138)
Supplement: Additional file 3 — MtDNA SSU rRNA gene sequences deposited at Genbank for fungi documented on Brazil nut. [file 1471-2180-14-138-S3.docx]

| **Species** | **Accession number** | **Strain type /Culture collection number** | **Reference** |
| --- | --- | --- | --- |
| *Acremonium sp.* | gb\|AY779325.1 | *SMH 2748* | Lumbsch,H.T., Schmitt,I., Lindemuth,R., Miller,A., Mangold,A., Fernandez,F. and Huhndorf,S. Performance of four ribosomal DNA regions to infer higher-level phylogenetic relationships of inoperculate euascomycetes (Leotiomyceta) Mol. Phylogenet. Evol. 34 (3), 512-524 (2005) |
| *Chaetomium thermophilum* | gb\|JX139037.1 | *UAMH 2024* | Hafez,M., Majer,A., Sethuraman,J., Rudski,S.M., Michel,F. and Hausner,G. The mtDNA rns gene landscape in the Ophiostomatales and other fungal taxa: Twintrons, introns, and intron-encoded proteins. Fungal Genet. Biol. 53, 71-83 (2013) |
| *Cladosporium sphaerospermum*  *Cladosporium cladosporioides* | gb\|DQ089641.1  gb\|AY291273.1 | *UPSC 957*  *ALI 50* | Zeng,Q.-Y. and Wang,X.-R. Rapid detection and quantification of Cladosporium in aerosols by real-time PCR. Unpublished  Zeng,Q.Y., Wang,X.R. and Blomquist,G. Development of mitochondrial SSU rDNA-based oligonucleotide probes for specific detection of common airborne fungi. Mol. Cell. Probes 17 (6), 281-288 (2003) |
| *Colletotrichum gloeosporioides* | gb\|HQ846820.1 | *OORC27* | Chowdappa,P., Chethana,C.S. and Madhura,S. Characterization of Colletotrichum gloeosporioides on different crops occurring in India based on six gene phylogenesis. Unpublished |
| *Exophiala nigra*  *Exophiala castellanii* | gb\|FJ225742.1  gb\|FJ225739.1 | *dH 12296*  *CBS 158.58* | Gueidan,C., Villasenor,C.R., de Hoog,G.S., Gorbushina,A.A., Untereiner,W.A. and Lutzoni,F. A rock-inhabiting ancestor for mutualistic and pathogen-rich fungal lineages. Stud. Mycol. 61, 111-119 (2008)  Gueidan,C., Villasenor,C.R., de Hoog,G.S., Gorbushina,A.A., Untereiner,W.A. and Lutzoni,F. A rock-inhabiting ancestor for mutualistic and pathogen-rich fungal lineages. Stud. Mycol. 61, 111-119 (2008) |
| *Fusarium oxysporum* | gb\|AF008472.1 | *NRRL 22550* | O'Donnell,K., Kistler,H.C., Cigelnik,E. and Ploetz,R.C. Multiple evolutionary origins of the fungus causing Panama disease of banana: concordant evidence from nuclear and mitochondrial gene genealogies. Proc. Natl. Acad. Sci. U.S.A. 95 (5), 2044-2049 (1998) |
| *Graphium putredinis* | gb\|FJ914747.1 | *HSAUP052348* | Zhang,T. and Pan,H. Molecular evolution of Doratomyces, Trichurus and Graphium species based on ITS and SSU rDNA sequences. Unpublished |
| *Paecilomyces variotii* | gb\|AY291281.1 | *UPSC 1651* | Zeng,Q.Y., Wang,X.R. and Blomquist,G. Development of mitochondrial SSU rDNA-based oligonucleotide probes for specific detection of common airborne fungi. Mol. Cell. Probes 17 (6), 281-288 (2003) |
| *Penicillium waksmanii*  *Penicillium punicae*  *Penicillium glabrum*  *Penicillium citrinum*  *Penicillium chrysogenum*  *Penicillium brevicompactum* | gb\|AF241472.1  gb\|AF241672.1  gb\|AF245271.1  gb\|AF003369.1  gb\|AY291284.1  gb\|AY291282.1 | KCTC6263  KCTC16055  KCTC10699  DAOM 216702  *UPSC 2020*  *ALI 319* | Hong,S.G., Park,Y.-D., Jeong,W. and Bae,K.S. Sequence comparison of mitochondrial small subunit ribosomal DNA in Penicillium. J. Microbiol. 38 (2), 62-65 (2000)  Park,Y.-D. and Bae,K.S. Phylogenetics of the fungi. Unpublished.  Park,Y.-D. and Bae,K.S. Phylogenetics of fungi. Unpublished.  Seifert,K.A. and Louis-Seize,G. Phylogeny and species concepts in the Penicillium aurantiogriseum complex as inferred from partial beta-tubulin gene DNA sequences. (in) Samson,R.A. and Pitt,J.I. (Eds.); INTEGRATION OF MODERN TAXONOMIC METHODS FOR PENICILLIUM AND ASPERGILLUS CLASSIFICATION: 189-198; Taylor and Francis Books Ltd., UK (2000).  Zeng,Q.Y., Wang,X.R. and Blomquist,G. Development of mitochondrial SSU rDNA-based oligonucleotide probes for specific detection of common airborne fungi. Mol. Cell. Probes 17 (6), 281-288 (2003).  Zeng,Q.Y., Wang,X.R. and Blomquist,G. Development of mitochondrial SSU rDNA-based oligonucleotide probes for specific detection of common airborne fungi. Mol. Cell. Probes 17 (6), 281-288 (2003) |
| *Phialophora verrucosa* | gb\|FJ225751.1 | AFTOL-ID 670 | Gueidan,C., Villasenor,C.R., de Hoog,G.S., Gorbushina,A.A., Untereiner,W.A. and Lutzoni,F. A rock-inhabiting ancestor for mutualistic and pathogen-rich fungal lineages. Stud. Mycol. 61, 111-119 (2008) |
| *Phoma sp.* | gb\|JQ238633.1 | *JDL-2012b 12S* | Lawrey,J.D., Diederich,P., Nelsen,M.P., Freebury,C., Sikaroodi,M. and Ertz,D. Phylogenetic placement of lichenicolous Phoma species in the Phaeosphaeriaceae (Pleosporales, Dothideomycetes). Fungal Divers. (2012) In press |
| *Rhizopus microsporus* | gb\|AY291255.1 |  | Zeng,Q.Y., Wang,X.R. and Blomquist,G. Development of mitochondrial SSU rDNA-based oligonucleotide probes for specific detection of common airborne fungi. Mol. Cell. Probes 17 (6), 281-288 (2003) |
| *Trichoderma reesei*  *Trichoderma harzianum*  *Trichoderma flavofuscum*  *Trichoderma pseudokoningii* | gb\|AY291279.1  gb\|AY755534.1  gb\|AF399190.1  gb\|AY291278.1 | QM 9414  T12  CBS 248.59  S-38 | Zeng,Q.Y., Wang,X.R. and Blomquist,G. Development of mitochondrial SSU rDNA-based oligonucleotide probes for specific detection of common airborne fungi. Mol. Cell. Probes 17 (6), 281-288 (2003).  Ergashev,K., Guzalova,A.G. and Leclerque,A. Molecular ribosomal phylogeny of entomopathogenic fungi from Uzbekistan.Unpublished  Kullnig-Gradinger,C.M., Szakacs,G. and Kubicek,C.P. Phylogeny ane evolution of the genus Trichoderma: a multigene approach. Mycol. Res. 106 (7), 757-767 (2002).  Zeng,Q.Y., Wang,X.R. and Blomquist,G. Development of mitochondrial SSU rDNA-based oligonucleotide probes for specific detection of common airborne fungi. Mol. Cell. Probes 17 (6), 281-288 (2003) |
| *Aspergillus aculeatus,* | gb\|EU982176.1 | ATHUM 5028 | Krimitzas,A., Pyrri,I., Kouvelis,V.N., Kapsanaki-Gotsi,E. and Typas,M.A. A phylogenetic analysis of greek isolates of Aspergillus species based on morphology and nuclear and mitochondrial gene sequences. Biomed Res Int 2013, 260395 (2013) |
| *Aspergillus aeneus,* | gb\|EU982174.1 | NRRL 4769 | Krimitzas,A., Pyrri,I., Kouvelis,V.N., Kapsanaki-Gotsi,E. and Typas,M.A. A phylogenetic analysis of greek isolates of Aspergillus species based on morphology and nuclear and mitochondrial gene sequences. Biomed Res Int 2013, 260395 (2013) |
| *Aspergillus awamori,* | gb\|EU982150.1 | ATHUM 5181 | Krimitzas,A., Pyrri,I., Kouvelis,V.N., Kapsanaki-Gotsi,E. and Typas,M.A. A phylogenetic analysis of greek isolates of Aspergillus species based on morphology and nuclear and mitochondrial gene sequences. Biomed Res Int 2013, 260395 (2013) |
| *Aspergillus bisporus,* | gb\|EU982166.1 | NRRL 3693 | Krimitzas,A., Pyrri,I., Kouvelis,V.N., Kapsanaki-Gotsi,E. and Typas,M.A. A phylogenetic analysis of greek isolates of Aspergillus species based on morphology and nuclear and mitochondrial gene sequences. Biomed Res Int 2013, 260395 (2013) |
| *Aspergillus brunneo-uniseriatus,* | gb\|EU982168.1 | NRRL 4273 | Krimitzas,A., Pyrri,I., Kouvelis,V.N., Kapsanaki-Gotsi,E. and Typas,M.A. A phylogenetic analysis of greek isolates of Aspergillus species based on morphology and nuclear and mitochondrial gene sequences. Biomed Res Int 2013, 260395 (2013) |
| *Aspergillus campestris,* | gb\|EU982163.1 | NRRL 13001 | Krimitzas,A., Pyrri,I., Kouvelis,V.N., Kapsanaki-Gotsi,E. and Typas,M.A. A phylogenetic analysis of greek isolates of Aspergillus species based on morphology and nuclear and mitochondrial gene sequences. Biomed Res Int 2013, 260395 (2013) |
| *Aspergillus clavatoflavus,* | gb\|EU982172.1 | NRRL 5113 | Krimitzas,A., Pyrri,I., Kouvelis,V.N., Kapsanaki-Gotsi,E. and Typas,M.A. A phylogenetic analysis of greek isolates of Aspergillus species based on morphology and nuclear and mitochondrial gene sequences. Biomed Res Int 2013, 260395 (2013) |
| *Aspergillus clavatus,*  *Aspergillus clavatus*  *Aspergillus clavatus genomic mtDNA* | gb\|EU982154.1  gb\|EU982155.1  gb\|JQ354999.1 | ATHUM 5032  ATHUM 5036  *NRRL1* | Krimitzas,A., Pyrri,I., Kouvelis,V.N., Kapsanaki-Gotsi,E. and Typas,M.A. A phylogenetic analysis of greek isolates of Aspergillus species based on morphology and nuclear and mitochondrial gene sequences. Biomed Res Int 2013, 260395 (2013).  Krimitzas,A., Pyrri,I., Kouvelis,V.N., Kapsanaki-Gotsi,E. and Typas,M.A. A phylogenetic analysis of greek isolates of Aspergillus species based on morphology and nuclear and mitochondrial gene sequences. Biomed Res Int 2013, 260395 (2013)  Joardar,V., Abrams,N.F., Hostetler,J., Paukstelis,P.J., Pakala,S., Pakala,S.B., Zafar,N., Abolude,O.O., Payne,G., Andrianopoulos,A., Denning,D.W. and Nierman,W.C. Sequencing of mitochondrial genomes of nine Aspergillus and Penicillium species identifies mobile introns and accessory genes as main sources of genome size variability. BMC Genomics 13 (1), 698 (2012) |
| *Aspergillus elongatus,* | gb\|EU982173.1 | NRRL 5176 | Krimitzas,A., Pyrri,I., Kouvelis,V.N., Kapsanaki-Gotsi,E. and Typas,M.A. A phylogenetic analysis of greek isolates of Aspergillus species based on morphology and nuclear and mitochondrial gene sequences. Biomed Res Int 2013, 260395 (2013) |
| *Aspergillus flavus,*  *Aspergillus flavus*  *Aspergillus flavus genomic mtDNA* | gb\|EU982151.1  gb\|EU982152.1  gb\|JQ355000.1 | ATHUM 5015  ATHUM 5033  *NRRL3357* | Krimitzas,A., Pyrri,I., Kouvelis,V.N., Kapsanaki-Gotsi,E. and Typas,M.A. A phylogenetic analysis of greek isolates of Aspergillus species based on morphology and nuclear and mitochondrial gene sequences. Biomed Res Int 2013, 260395 (2013)  Joardar,V., Abrams,N.F., Hostetler,J., Paukstelis,P.J., Pakala,S., Pakala,S.B., Zafar,N., Abolude,O.O., Payne,G., Andrianopoulos,A., Denning,D.W. and Nierman,W.C. Sequencing of mitochondrial genomes of nine Aspergillus and Penicillium species identifies mobile introns and accessory genes as main sources of genome size variability. BMC Genomics 13 (1), 698 (2012) |
| *Aspergillus fumigatus,*  *Aspergillus fumigatus,*  *Aspergillus fumigatus genomic mtDNA*  *Aspergillus fumigatus genomic mtDNA*  *Aspergillus fumigatus genomic mtDNA* | gb\|AY291258.1  gb\|EU982153.1  gb\|JQ346807.1  gb\|JQ346808.1  gb\|JQ346809.1 | UPSC 1771  ATHUM 5013  *A1163*  *AF293*  *AF210* | Zeng,Q.Y., Wang,X.R. and Blomquist,G. Development of mitochondrial SSU rDNA-based oligonucleotide probes for specific detection of common airborne fungi. Mol. Cell. Probes 17 (6), 281-288 (2003)  Krimitzas,A., Pyrri,I., Kouvelis,V.N., Kapsanaki-Gotsi,E. and Typas,M.A. A phylogenetic analysis of greek isolates of Aspergillus species based on morphology and nuclear and mitochondrial gene sequences. Biomed Res Int 2013, 260395 (2013)  Joardar,V., Abrams,N.F., Hostetler,J., Paukstelis,P.J., Pakala,S., Pakala,S.B., Zafar,N., Abolude,O.O., Payne,G., Andrianopoulos,A., Denning,D.W. and Nierman,W.C. Sequencing of mitochondrial genomes of nine Aspergillus and Penicillium species identifies mobile introns and accessory genes as main sources of genome size variability. BMC Genomics 13 (1), 698 (2012) |
| *Aspergillus giganteus,* | gb\|EU982181.1 | NRRL 10 | Krimitzas,A., Pyrri,I., Kouvelis,V.N., Kapsanaki-Gotsi,E. and Typas,M.A. A phylogenetic analysis of greek isolates of Aspergillus species based on morphology and nuclear and mitochondrial gene sequences. Biomed Res Int 2013, 260395 (2013) |
| *Aspergillus janus,* | gb\|EU982175.1 | NRRL 1787 | Krimitzas,A., Pyrri,I., Kouvelis,V.N., Kapsanaki-Gotsi,E. and Typas,M.A. A phylogenetic analysis of greek isolates of Aspergillus species based on morphology and nuclear and mitochondrial gene sequences. Biomed Res Int 2013, 260395 (2013) |
| *Aspergillus kambarensis,* | gb\|U29219.1 | NRRL 3751 | Peterson,S.W. Phylogenetic analysis of Aspergillus based upon rDNA sequence. National Center for Agricultural Utilization Research, Microbial Properties Research, 1815 N. University St., Peoria, IL 61604, USA |
| *Aspergillus leporis,* | gb\|U29228.1 | NRRL 6599 | Peterson,S.W. Phylogenetic analysis of Aspergillus based upon rDNA sequence. National Center for Agricultural Utilization Research, Microbial Properties Research, 1815 N. University St., Peoria, IL 61604, USA |
| *Aspergillus niger,*  *Aspergillus niger,*  *Aspergillus niger,*  *Aspergillus niger genomic mtDNA* | gb\|AY291253.1  gb\|EU982148.1  gb\|EU982149.1  gb\|DQ207726.1 | UPSC 1769  ATHUM 5044  ATHUM 2539  *N909* | Zeng,Q.Y., Wang,X.R. and Blomquist,G. Development of mitochondrial SSU rDNA-based oligonucleotide probes for specific detection of common airborne fungi. Mol. Cell. Probes 17 (6), 281-288 (2003)  Krimitzas,A., Pyrri,I., Kouvelis,V.N., Kapsanaki-Gotsi,E. and Typas,M.A. A phylogenetic analysis of greek isolates of Aspergillus species based on morphology and nuclear and mitochondrial gene sequences. Biomed Res Int 2013, 260395 (2013)  Juhasz,A., Pfeiffer,I., Keszthelyi,A., Kucsera,J., Vagvolgyi,C. and Hamari,Z. Comparative analysis of the complete mitochondrial genomes of Aspergillus niger mtDNA type 1a and Aspergillus tubingensis mtDNA type 2b. FEMS Microbiol. Lett. 281 (1), 51-57 (2008) |
| *Aspergillus niveus,* | gb\|EU982164.1 | ATHUM 5029 | Krimitzas,A., Pyrri,I., Kouvelis,V.N., Kapsanaki-Gotsi,E. and Typas,M.A. A phylogenetic analysis of greek isolates of Aspergillus species based on morphology and nuclear and mitochondrial gene sequences. Biomed Res Int 2013, 260395 (2013) |
| *Aspergillus nomius,* | gb\|U29227.1 | NRRL 3353 | Peterson,S.W. Phylogenetic analysis of Aspergillus based upon rDNA sequence. Unpublished |
| *Aspergillus ochraceus,*  *Aspergillus ochraceus* | gb\|AY291267.1  gb\|EU982177.1 | UPSC 1983  ATHUM 5014 | Zeng,Q.Y., Wang,X.R. and Blomquist,G. Development of mitochondrial SSU rDNA-based oligonucleotide probes for specific detection of common airborne fungi. Mol. Cell. Probes 17 (6), 281-288 (2003)  Krimitzas,A., Pyrri,I., Kouvelis,V.N., Kapsanaki-Gotsi,E. and Typas,M.A. A phylogenetic analysis of greek isolates of Aspergillus species based on morphology and nuclear and mitochondrial gene sequences. Biomed Res Int 2013, 260395 (2013) |
| *Aspergillus oryzae,*  *Aspergillus oryzae genomic mtDNA* | gb\|EU982162.1  gb\|AP007176 | ATHUM 4958  *RIB40* | Krimitzas,A., Pyrri,I., Kouvelis,V.N., Kapsanaki-Gotsi,E. and Typas,M.A. A phylogenetic analysis of greek isolates of Aspergillus species based on morphology and nuclear and mitochondrial gene sequences. Biomed Res Int 2013, 260395 (2013)  Machida,M. et al. Genome sequencing and analysis of Aspergillus oryzae. Nature 438 (7071), 1157-1161 (2005) |
| *Aspergillus parasiticus,*  *Aspergillus parasiticus* | gb\|EU982160.1  gb\|EU982161.1 | ATHUM 5037  ATHUM 5038 | Krimitzas,A., Pyrri,I., Kouvelis,V.N., Kapsanaki-Gotsi,E. and Typas,M.A. A phylogenetic analysis of greek isolates of Aspergillus species based on morphology and nuclear and mitochondrial gene sequences. Biomed Res Int 2013, 260395 (2013) |
| *Aspergillus penicillioides,* | gb\|AY291264.1 | ALI 231 | Zeng,Q.Y., Wang,X.R. and Blomquist,G. Development of mitochondrial SSU rDNA-based oligonucleotide probes for specific detection of common airborne fungi. Mol. Cell. Probes 17 (6), 281-288 (2003) |
| *Aspergillus puniceus,* | gb\|EU982159.1 | ATHUM 5434 | Krimitzas,A., Pyrri,I., Kouvelis,V.N., Kapsanaki-Gotsi,E. and Typas,M.A. A phylogenetic analysis of greek isolates of Aspergillus species based on morphology and nuclear and mitochondrial gene sequences. Biomed Res Int 2013, 260395 (2013) |
| *Aspergillus restrictus,* | gb\|EU982167.1 | NRRL 154 | Krimitzas,A., Pyrri,I., Kouvelis,V.N., Kapsanaki-Gotsi,E. and Typas,M.A. A phylogenetic analysis of greek isolates of Aspergillus species based on morphology and nuclear and mitochondrial gene sequences. Biomed Res Int 2013, 260395 (2013) |
| *Aspergillus rubrum,* | gb\|EU982158.1 | ATHUM 5183 | Krimitzas,A., Pyrri,I., Kouvelis,V.N., Kapsanaki-Gotsi,E. and Typas,M.A. A phylogenetic analysis of greek isolates of Aspergillus species based on morphology and nuclear and mitochondrial gene sequences. Biomed Res Int 2013, 260395 (2013) |
| *Aspergillus sclerotiorum,* | gb\|EU982178.1 | NRRL 415 | Krimitzas,A., Pyrri,I., Kouvelis,V.N., Kapsanaki-Gotsi,E. and Typas,M.A. A phylogenetic analysis of greek isolates of Aspergillus species based on morphology and nuclear and mitochondrial gene sequences. Biomed Res Int 2013, 260395 (2013) |
| *Aspergillus silvaticus,* | gb\|AY291266.1 | ALI 234 | Zeng,Q.Y., Wang,X.R. and Blomquist,G. Development of mitochondrial SSU rDNA-based oligonucleotide probes for specific detection of common airborne fungi. Mol. Cell. Probes 17 (6), 281-288 (2003) |
| *Aspergillus sojae,* | gb\|U29216.1 | NRRL 1988 | Peterson,S.W. Phylogenetic analysis of Aspergillus based upon rDNA sequence. Unpublished |
| *Aspergillus subolivaceus,* | gb\|U29222.1 | NRRL 4998 | Peterson,S.W. Phylogenetic analysis of Aspergillus based upon rDNA sequence. National Center for Agricultural Utilization Research, Microbial Properties Research, 1815 N. University St., Peoria, IL 61604, USA |
| *Aspergillus sydowii,* | gb\|EU982169.1 | ATHUM 5093 | Krimitzas,A., Pyrri,I., Kouvelis,V.N., Kapsanaki-Gotsi,E. and Typas,M.A. A phylogenetic analysis of greek isolates of Aspergillus species based on morphology and nuclear and mitochondrial gene sequences. Biomed Res Int 2013, 260395 (2013) |
| *Aspergillus tamarii,* | gb\|U29224.1 | NRRL 20818 | Peterson,S.W. Phylogenetic analysis of Aspergillus based upon rDNA sequence. Unpublished |
| *Aspergillus terreus,*  *Aspergillus terreus genomic mtDNA* | gb\|EU982165.1  gb\|JQ355001.1 | ATHUM 4761  *NIH2624* | Krimitzas,A., Pyrri,I., Kouvelis,V.N., Kapsanaki-Gotsi,E. and Typas,M.A. A phylogenetic analysis of greek isolates of Aspergillus species based on morphology and nuclear and mitochondrial gene sequences. Biomed Res Int 2013, 260395 (2013)  Joardar,V., Abrams,N.F., Hostetler,J., Paukstelis,P.J., Pakala,S., Pakala,S.B., Zafar,N., Abolude,O.O., Payne,G., Andrianopoulos,A., Denning,D.W. and Nierman,W.C. Sequencing of mitochondrial genomes of nine Aspergillus and Penicillium species identifies mobile introns and accessory genes as main sources of genome size variability. BMC Genomics 13 (1), 698 (2012) |
| *Aspergillus terricola,* | gb\|U29212.1 | NRRL 426 | Peterson,S.W. Phylogenetic analysis of Aspergillus based upon rDNA sequence. National Center for Agricultural Utilization Research, Microbial Properties Research, 1815 N. University St., Peoria, IL 61604, USA |
| *Aspergillus thomii,* | gb\|U29217.1 | NRRL 2097 | Peterson,S.W. Phylogenetic analysis of Aspergillus based upon rDNA sequence. National Center for Agricultural Utilization Research, Microbial Properties Research, 1815 N. University St., Peoria, IL 61604, USA |
| *Aspergillus ustus,*  *Aspergillus ustus,* | gb\|EU982170.1  gb\|EU982171.1 | ATHUM 5097  ATHUM 5103 | Krimitzas,A., Pyrri,I., Kouvelis,V.N., Kapsanaki-Gotsi,E. and Typas,M.A. A phylogenetic analysis of greek isolates of Aspergillus species based on morphology and nuclear and mitochondrial gene sequences. Biomed Res Int 2013, 260395 (2013) |
| *Aspergillus versicolor.*  *Aspergillus versicolor* | gb\|AY291275.1  gb\|EU982182.1 | UPSC 2027  ATHUM 2541 | Zeng,Q.Y., Wang,X.R. and Blomquist,G. Development of mitochondrial SSU rDNA-based oligonucleotide probes for specific detection of common airborne fungi. Mol. Cell. Probes 17 (6), 281-288 (2003)  Krimitzas,A., Pyrri,I., Kouvelis,V.N., Kapsanaki-Gotsi,E. and Typas,M.A. A phylogenetic analysis of greek isolates of Aspergillus species based on morphology and nuclear and mitochondrial gene sequences. Biomed Res Int 2013, 260395 (2013) |
| *Eurotium herbariorum* | gb\|AY291259.1 | ALI 216 | Zeng,Q.Y., Wang,X.R. and Blomquist,G. Development of mitochondrial SSU rDNA-based oligonucleotide probes for specific detection of common airborne fungi. Mol. Cell. Probes 17 (6), 281-288 (2003) |
| *Eurotium rubrum* | gb\|AF346424.1 | CBS 530.65 | Lindemuth,R., Wirtz,N. and Lumbsch,H.T. Phylogenetic analysis of nuclear and mitochondrial rDNA sequences supports the view that loculoascomycetes (Ascomycota) are not monophyletic. Mycol. Res. 105 (10), 1176-1181 (2001) |
